# GATA2 controls alveolar macrophage inflammatory gene expression and metabolic function

**DOI:** 10.1172/jci.insight.196246

**Published:** 2026-02-19

**Authors:** Morgan Jackson-Strong, Satarupa Ganguly, Aaron Francis, Flavia Rago, Jitendra Kanshana, Brandon A. Michalides, Lihong Teng, Omkar S. Betsur, Sonia Kruszelnicki, Karsen E. Shoger, Aaron Kim, Kay Bajpai, Amina Suleyman, Abigail Sekyere, Mika Hara, Varsha Sriram, Alok Kumar, Greg M. Delgoffe, Niranjana Natarajan, John F. Alcorn, Alison B. Kohan, Rachel A. Gottschalk

**Affiliations:** 1 Department of Immunology, University of Pittsburgh School of Medicine, Pittsburgh, Pennsylvania, USA.; 2 Center for Systems Immunology, University of Pittsburgh, Pittsburgh, Pennsylvania, USA.; 3 Division of Pulmonary Medicine, Department of Pediatrics, UPMC Children’s Hospital of Pittsburgh, Pittsburgh, Pennsylvania, USA.; 4 Department of Medicine, Division of Endocrinology and Metabolism, and; 5 Division of Rheumatology and Clinical Immunology, Department of Medicine, University of Pittsburgh School of Medicine, Pittsburgh, Pennsylvania, USA.; 6 Tumor Microenvironment Center, UPMC Hillman Cancer Institute, Pittsburgh, Pennsylvania, USA.

**Keywords:** Immunology, Pulmonology, Macrophages, Transcription

## Abstract

Alveolar macrophages (AMs) catabolize lipid-rich pulmonary surfactant to support gas exchange and have antiinflammatory programming to limit tissue damage in response to minor challenges. GATA transcription factors (TFs) shape immune cell fates, and GATA2 is expressed in a lung-specific manner in macrophages. GATA2 mutations and lung macrophage downregulation of GATA2 have been associated with chronic pulmonary pathologies in humans, but the role of GATA2 in coordinating AM function is not well defined. Using mice with myeloid-specific deletion of the GATA2 DNA binding C-terminal zinc finger domain, we show that GATA2 deficiency promotes enhanced inflammatory gene expression and metabolic dysfunction in AMs in response to type 2 stimuli. Although homeostatic functions of AMs remain largely intact, GATA2 deficiency increases expression of type 2 response genes during IL-33–induced inflammation. Coincident with GATA2-dependent expression of genes in metabolic pathways, Seahorse metabolic flux analysis indicates that AM metabolism is compromised in the absence of GATA2. AM GATA2-dependent gene networks are enriched for targets of TFs previously demonstrated to interact with GATA2 in other cellular contexts, including PU.1, PPARγ, and other regulators of AM function. Our data suggest that GATA2 modulates AM metabolic and transcriptomic programming to restrain responses and maintain AM identity during inflammation.

## Abstract 2

The transcription factor GATA2 is associated with chronic pulmonary pathologies. We show that GATA2 deficiency promotes mitochondrial dysfunction and profibrotic gene expression in lung macrophages during inflammation.

## Introduction

Tissue-resident macrophages perform specialized functions tailored to the unique requirements of the specific microenvironment. Alveolar macrophages (AMs) have a critical role in maintaining lung homeostasis; they degrade surfactant lipids formed by alveolar epithelial cells to support gas exchange and have steady-state, antiinflammatory functions to limit lung tissue damage in response to minor challenge (1, 2). AMs are capable of shifting their functional programs in response to environmental stimuli and play an important role in host defense (3). However, AM inflammatory responses are generally more constrained than monocyte-derived macrophages in the same inflammatory contexts; using BM chimeras to distinguish tissue-resident and monocyte-derived AMs, studies show that tissue-resident AMs have reduced transcriptomic changes in response to bleomycin-induced fibrosis or influenza infection (4, 5).

Macrophage metabolic programming is dynamic and responsive to extrinsic cues, and metabolic reprogramming underlies the ability of macrophages to switch inflammatory functions (6). AM lipid metabolism has been linked to the unique activation profiles of AMs; BM-derived macrophages (BMDMs) and peritoneal macrophages rely on glycolysis for inflammatory responses, whereas AMs are less dependent on this pathway in response to both type 1 and type 2 inflammatory stimuli (7, 8). The homeostatic lung cytokines, GM-CSF and TGF-β, shape the development and functions of AMs in part through PU.1- and PPAR-mediated regulation of AM lipid metabolism (9, 10). Of note, dysregulated macrophage metabolism has been associated with lung pathology, with AM metabolic defects being mechanistically linked to pulmonary fibrosis (11–13).

We recently identified GATA2 as a GM-CSF–regulated, lung-specific transcription factor (TF) in macrophages. In the absence of the SOCS protein CISH, AMs exhibited increased expression of GATA2 and its target genes (14), consistent with CISH acting as a negative regulator of STAT5 and GATA2 being a STAT5 target (15, 16). Increased GATA2 expression was coincident with a foamy, lipid-laden AM phenotype and differential expression of genes in lipid processing pathways. Of note, mutations in GATA2 are associated with the development of pulmonary alveolar proteinosis, a disease characterized by an accumulation of surfactant in the lungs (17–19).

GATA2 has been extensively studied in hematopoiesis and adipocyte differentiation, orchestrating these processes through interactions with TFs that are also key regulators of AM function and lipid metabolism. During adipocyte differentiation, GATA2 interfaces with PPARγ, CEBPβ, and CEBP*α* (20, 21); these TFs are highly expressed in AMs, with PPARγ and CEBPβ controlling critical genes for surfactant catabolism and lipid metabolism in AMs, and CEBPα regulating mitochondrial respiration in other contexts (22–25). PU.1, also essential for AM identity and function (26), has well-described interactions with GATA2 during differentiation, with antagonistic or cooperative interplay depending on the cell lineage (27). Although few studies have investigated GATA2 function in macrophages, reports have linked GATA2 to phagocytic abilities of AMs and macrophage dysfunction in atherosclerosis (28, 29). Recent analysis of human lung single-cell RNA-seq (scRNA-seq) data revealed decreased *Gata2* expression in macrophages in patients with chronic obstructive pulmonary disease (COPD), findings that were recapitulated in a cigarette smoke–induced COPD mouse model, consistent with GATA2-dependent inflammatory functions in vitro (30). While this was the first report, to our knowledge, linking GATA2 to inflammatory functions, but GATA3 has been implicated in promoting metabolic reprogramming that controls macrophage alternative activation and tissue-repair programs in response to IL-33 (31). Previous mechanistic efforts to define GATA2 function in the regulation of lung macrophages have relied on *Gata2* knockdown or overexpression in vitro, limiting our ability to define the interplay between GATA2 and other regulatory TFs in response to complex homeostatic and inflammatory cues in the lung microenvironment.

We generated a murine model with myeloid-specific GATA2 deficiency and evaluated the role of GATA2 in AM homeostatic functions and responsiveness to inflammatory stimuli. Our data suggest that GATA2 is not required for AM development, maintenance, or homeostatic lipid uptake, but may play a role in regulating phagocytic cargo processing. In response to IL-33–induced inflammation, GATA2-deficient AMs demonstrated increased expression of metabolic pathway genes and genes associated with type 2 responses, many of which were known targets of GATA2-interacting TFs, including PU.1 and PPARγ. Collectively, our findings suggest that GATA2 supports lung-specific macrophage programming by helping to constrain AM metabolic and inflammatory responses, providing mechanistic insight into how GATA2 mutations and decreased *Gata2* expression may contribute to pathology in chronic lung diseases.

## Results

### AM GATA2 expression is context dependent.

**Figure 1:**
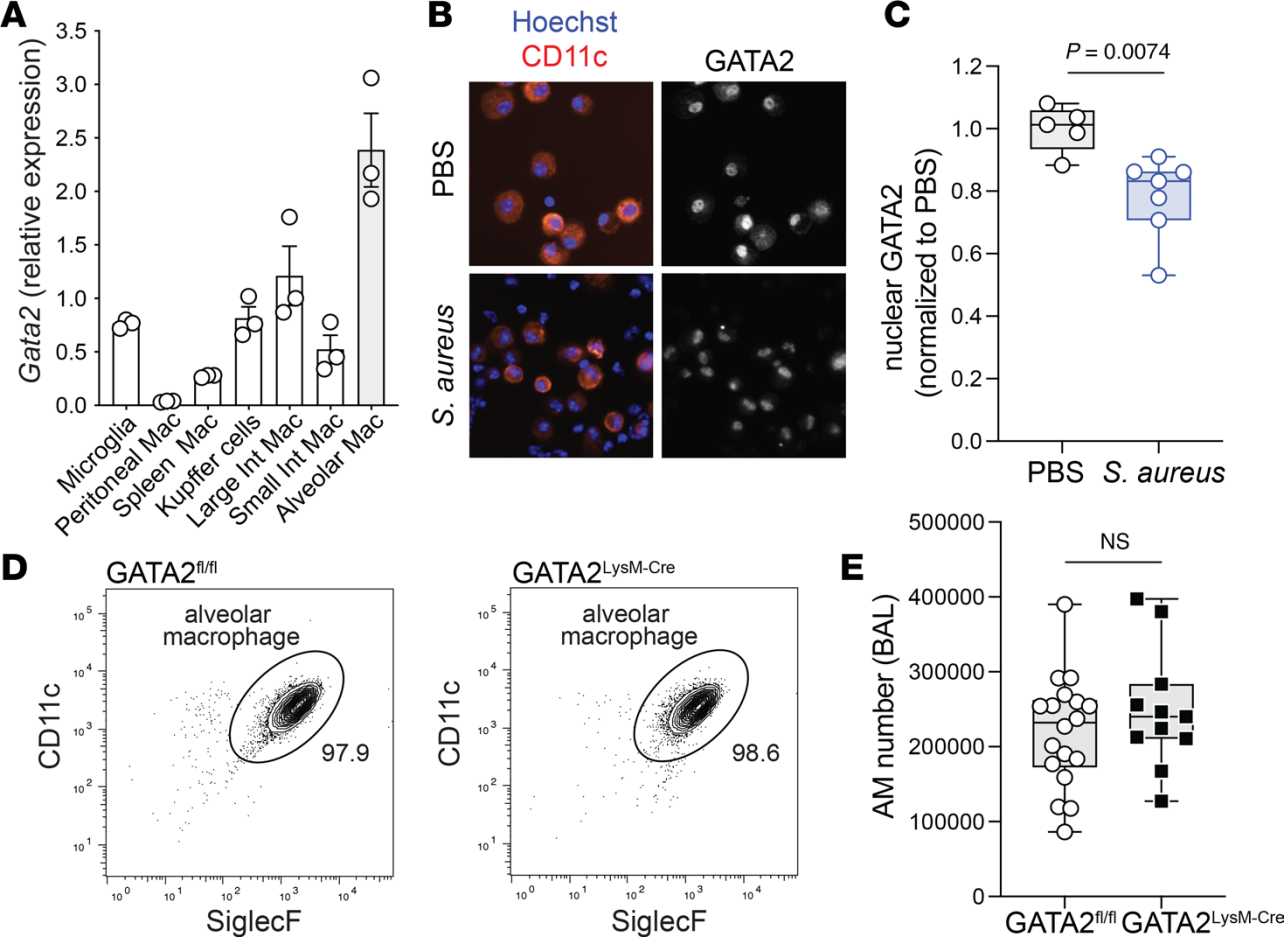
Alveolar macrophage GATA2 expression is context dependent. (**A**) Reanalysis of RNA-seq data from Qie et al. (32), quantifying relative expression of the *Gata2* gene in tissue-resident macrophages (*n* = 3; mean ± SEM). (**B** and **C**) WT mice (C57BL/6) were intranasally infected with *S*. *aureus* for 24 hours, and AMs were isolated using bronchoalveolar lavage (BAL). (**B**) Representative images showing total GATA2 protein, CD11c, and Hoechst nuclear stain. (**C**) The MFI of nuclear total GATA2 was quantified in CD11c^+^ cells. Flow cytometry gating strategy (**D**) and quantification (**E**) of steady-state CD11c^+^SiglecF^+^ AMs isolated by BAL. Points represent individual mice pooled from 2 independent experiments (**C**) and 5 independent experiments (**E**). Significance was determined using unpaired 2-tailed *t* test.

In agreement with our previous report (14), analysis of published tissue-resident macrophage RNA-seq data showed that macrophage expression of *Gata2* was higher in the lung compared with other tissues (Figure 1A) (32). Lung-specific expression of GATA2 was consistent with the induction of *Gata2* by GM-CSF (Supplemental Figure 1A; supplemental material available online with this article; https://doi.org/10.1172/jci.insight.196246DS1) and reports that the GATA2 gene is directly regulated by STAT5 (16). The relative expression of the GATA2 gene in AMs was low compared with TFs known to shape AM identity and function (Supplemental Figure 1B), likely limiting its detection in macrophages in mouse and human scRNA-seq datasets. Despite low mRNA abundance, GATA2 protein could be detected in the nucleus of CD11c^+^ AMs isolated by bronchoalveolar lavage (BAL) (Figure 1B). Although we had previously reported an increase in GATA2 expression and activity in CISH-deficient mice (14), we found that GATA2 protein was decreased in AMs after intranasal infection with *Staphylococcus* *aureus* (Figure 1, B and C); we note that GATA2 was not detected in recruited CD11c^–^ BAL cells (Figure 1B). Our results are consistent with other studies reporting AM GATA2 downregulation during lung inflammation: GATA2 expression was reported in AMs, resident lung monocytes, and bronchial epithelial cells in the rat lung, and expression was downregulated during *P*. *carinii* infection (28); lung macrophage expression of the GATA2 gene was decreased in patients with COPD and people who smoke (30); and GATA2 expression was decreased in AMs isolated from mice with cigarette smoke–induced COPD (30). Most recently, GATA2 was identified as a target of the lncRNA ROCKI in macrophages (33); given that this lncRNA is increased downstream of inflammatory signals, this provides one potential mechanism for downregulation of GATA2 in inflammatory contexts.

To investigate the role of GATA2 in AMs, we generated myeloid-specific *Gata2*-deficient mice by crossing GATA2^fl/fl^ mice (34) with LysM-Cre mice (35) to create GATA2^LysM-Cre^ mice. This design deletes the DNA-binding C-terminal zinc finger domain of GATA2 in the presence of myeloid-specific Cre recombinase (Supplemental Figure 1B). We quantified steady-state CD11c^+^SiglecF^+^ AMs in the BAL by flow cytometry and found that AM numbers did not significantly differ between GATA2^fl/fl^ and GATA2^LysM-Cre^ mice (Figure 1, D and E). Thus, macrophage expression of GATA2 is regulated by the lung microenvironment and is not required for the development and maintenance of AMs.

### Homeostatic functions remain largely intact in GATA2-deficient AMs.

**Figure 2:**
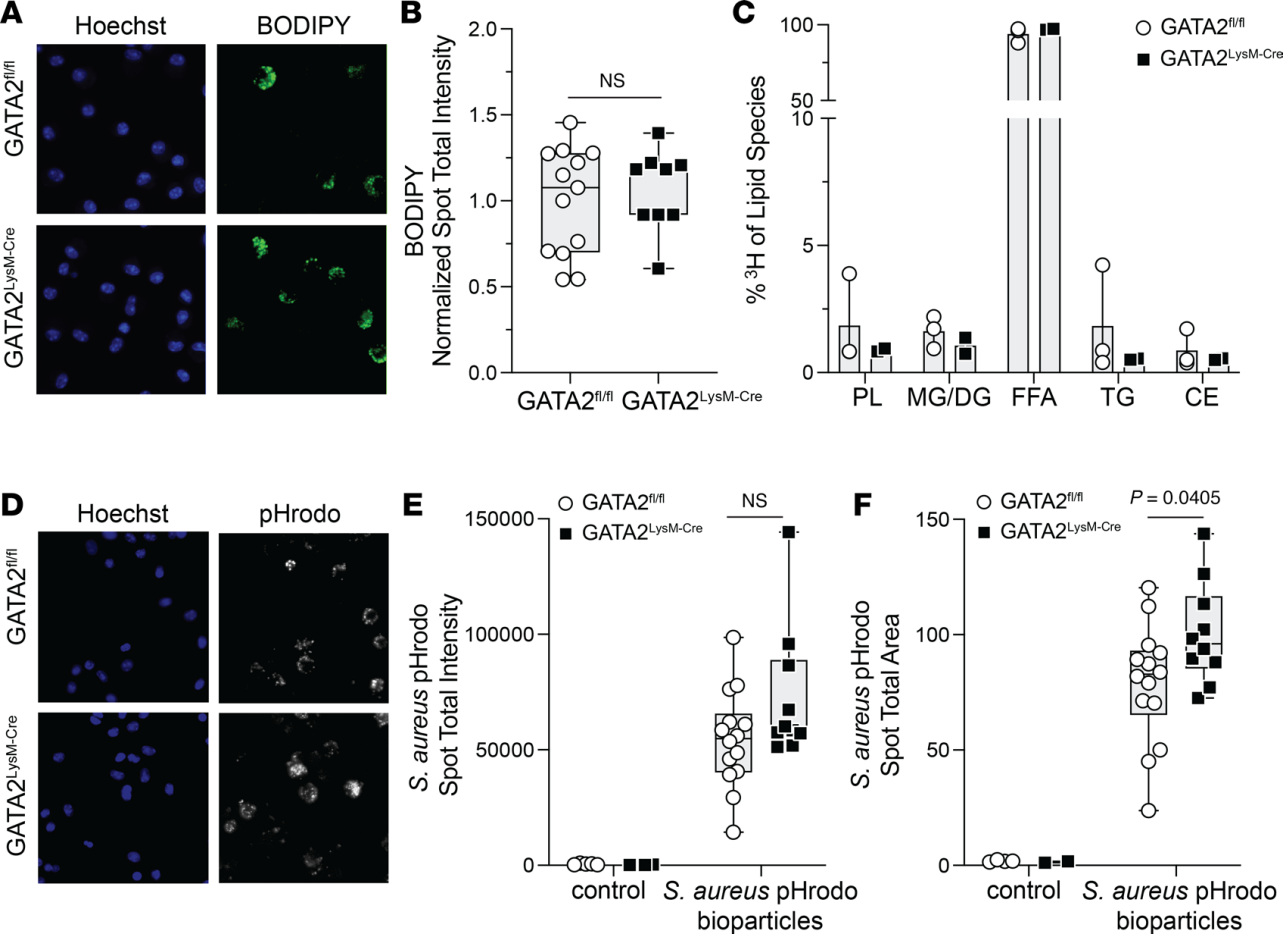
Homeostatic functions remain largely intact in GATA2-deficient alveolar macrophages. GATA2^fl/fl^ and GATA2^LysM-Cre^ AMs were isolated using BAL. (**A** and **B**) Neutral lipids were stained directly ex vivo using BODIPY dye. (**A**) Representative images of BODIPY and Hoechst nuclear stain. (**B**) Quantification of BODIPY spot total intensity; points represent individual mice pooled from 3 independent experiments, normalized based on the average GATA2^fl/fl^ per experiment. (**C**) The percentage of ^3^H-labeled lipid species quantified via TLC after Folch lipid extraction; points represent individual mice and are representative of 2 independent experiments. (**D**–**F**) AMs were treated with *S*. *aureus* pHrodo bioparticles for 90 minutes and, after fixation, the spot total intensity of internalized bioparticles was quantified via immunofluorescence imaging. (**D**) Representative images of internalized pHrodo bioparticles and Hoechst nuclear stain. (**E** and **F**) Spot total intensity and spot total area of pHrodo-labeled bioparticles; points represent individual mice and are pooled from 5 independent experiments. Significance was determined using unpaired 2-tailed *t* test.

Given the central role of surfactant catabolism in AM lung-specific function, which depends on tightly regulated lipid metabolism, we examined whether GATA2 deficiency disrupts lipid homeostasis in AMs at steady state. Using AMs isolated directly ex vivo via BAL, we used BODIPY dye to stain intracellular neutral lipids and quantified the spot total intensity in CD11c^+^ AMs. We found no significant difference in neutral lipid content in GATA2-deficient AMs compared with GATA2^fl/fl^ controls (Figure 2, A and B). We further assessed AM homeostatic functions by measuring the acute lipid uptake of dipalmitoyl-phosphatidylcholine (DPPC), the major lipid species found in pulmonary surfactant. AMs were treated ex vivo with NBD (7-nitro-2,1,3-benzoxadiazol-4-yl)–labeled (20 ng/mL; diluted in pulmonary surfactant) for 30 minutes, and no significant difference in DPPC uptake was observed in GATA2-deficient AMs (Supplemental Figure 1C). To determine whether GATA2^fl/fl^ and GATA2^LysM-Cre^ AMs differed in their lipid processing, we incubated serum-starved AMs with radiolabeled fatty acids (^3^H-oleic acid) diluted in pulmonary surfactant and performed a Folch lipid extraction to isolate the lipids and separate the different lipid species using TLC. Upon identifying and quantifying triglycerides, diacylglycerols, monoacylglycerols, free fatty acids (FFAs), and phospholipids, we found that the ability of GATA2-deficient AM processing of FFA into the various lipid species was comparable to that of GATA2^fl/fl^ AMs (Figure 2C). Notably, most of the radiolabeled FFAs were not converted to other lipid species. Together, these data suggest that GATA2 deficiency does not substantially affect AM homeostatic functions, including neutral lipid content, lipid uptake, or lipid processing under steady-state conditions.

We next assessed AM phagocytic processing of pHrodo-labeled *S*. *aureus* bioparticles ex vivo. The fluorescence of pHrodo dye is sensitive to pH, with fluorescence being activated in acidic environments such as lysosomes. After 90 minutes of bioparticle exposure, we quantified pHrodo spot total intensity and spot total area within CD11c^+^ AMs and found increased pHrodo in GATA2-deficient AMs compared with control AMs, reaching statistical significance for the spot total area quantification metric (Figure 2, D–F). We also compared GATA2^fl/fl^ and GATA2^LysM-Cre^ AM phagocytosis of *S*. *aureus* bioparticles labeled with Alexa Fluor 594 and or of yellow-green carboxylate-modified polystyrene beads, both independent of lysosomal acidification, and observed no significant difference in uptake at 90 minutes (Supplemental Figure 1, D–G). Thus, although GATA2-deficient AMs largely maintain homeostatic functions at baseline, we observed selective alterations in phagocytic cargo handling, indicating that GATA2 may influence specific functional processes. Our *S*. *aureus* pHrodo results align with prior findings in THP1 cells demonstrating that GATA2 overexpression impairs phagosome-lysosome fusion and suppresses phagosomal acidification (29), suggesting a role for GATA2 in regulating phagocytic cargo processing.

### GATA2 deficiency is associated with reduced AM numbers in bleomycin-induced fibrosis.

**Figure 3:**
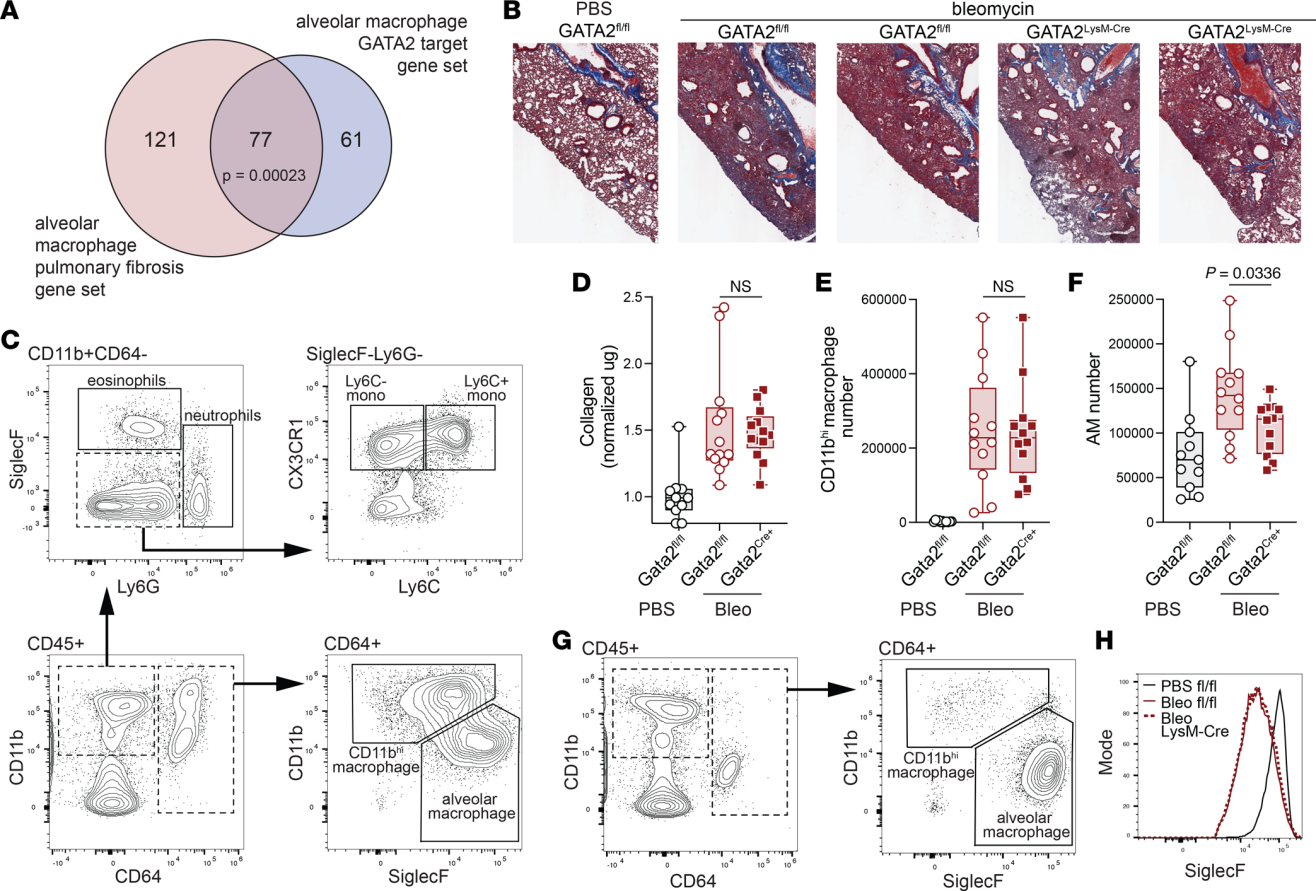
GATA2-deficiency is associated with reduced alveolar macrophage numbers in bleomycin-induced fibrosis. (**A**) Venn diagram represents the overlap between AM pulmonary fibrosis gene set and AM GATA2 target gene set. (**B**–**H**) Mice were treated intratracheally with bleomycin (3 U/kg) and euthanized 14 days later for lung tissue harvest and analysis. (**B**) Representative Masson trichrome stain. (**C**) Representative bleomycin lung flow cytometry plots showing gating strategy for eosinophils, neutrophils, monocytes, and macrophages. (**D**) Soluble collagen was quantified by Sircol assay. Quantification of CD11b^hi^ macrophages (**E**) and SiglecF^+^CD11b^lo^ AMs (**F**). (**G**) Representative PBS lung flow cytometry plots. (**H**) Flow cytometry histogram showing AM SiglecF expression in representative PBS- or bleomycin-treated mice. Points represent individual mice pooled from 3 independent experiments; collagen data in **D** were normalized based on the average PBS control sample per experiment. Significance was determined using 1-way ANOVA with post hoc pairwise testing using Šídák’s multiple-comparison test.

Lung macrophage expression of GATA2 decreases in patients with COPD and in mice with cigarette smoke–induced COPD, and studies using knockdown and overexpression in RAW264.7 macrophages demonstrate that GATA2 regulates macrophage expression of *Mmp9* and *Mmp12*, as well as inflammatory chemokine genes and other genes associated with profibrotic lung inflammation (30). We examined the overlap between fibrosis-associated genes and known GATA2 targets by leveraging AM and pulmonary fibrosis gene sets from Harmonizome 3.0 to define an “AM + Pulmonary Fibrosis” gene list (see Methods). We also defined an “AM + GATA2 targets” gene list, composed of known GATA2 targets from the ChEA3 TF target database (https://maayanlab.cloud/chea3/) within the same AM gene set. We then determined the intersection of these 2 lists and assessed the significance of the observed overlap (Figure 3A). Using a 1-sided Fisher’s exact test, we found that GATA2 target genes were significantly enriched in the AM + Pulmonary Fibrosis set (*P* value = 0.00023), suggesting that GATA2 could play a functional role in regulating fibrosis-associated type 2 inflammatory responses in AMs.

To assess the role of GATA2 in lung fibrosis progression, we used a model of intratracheal bleomycin administration (3 U/kg) to induce fibrosis over 14 days (Figure 3B). At day 14, mice were euthanized and lungs were harvested for analysis of immune populations and quantification of soluble collagen (Figure 3, C and D). Although collagen deposition trended higher in GATA2-deficient animals (Figure 3D), significant GATA2-dependent differences were not observed in collagen or in the abundance of infiltrating immune populations (Supplemental Figure 2). Flow cytometric analysis of bleomycin-treated mice revealed 2 CD64^+^ macrophage populations, consisting of CD11b^hi^ macrophages and CD11b^lo^ SiglecF^+^ AMs (Figure 3, C, E, and F). We observed a significant decrease in AMs in GATA2^LysM-Cre^ mice compared with GATA2^fl/fl^ controls (Figure 3F). Notably, these AMs exhibited substantially reduced SiglecF expression compared with AMs from PBS-treated mice (Figure 3, G and H). We speculate that lower SiglecF expression indicates replacement of tissue-resident AMs by monocyte-derived AMs, which have been shown to express lower SiglecF using a lineage-tracing system in the context of bleomycin-induced fibrosis (5). However, we acknowledge that downregulation of SiglecF on tissue-resident AMs has also been reported during lung inflammation (36), and therefore SiglecF expression alone cannot definitively distinguish macrophage origin in this setting. Thus, GATA2 may contribute to AM survival during inflammation or to monocyte-derived AM differentiation.

### IL-33 indirectly induces a robust AM type 2 transcriptomic program.

Given the altered AM phenotype that we observed 14 days after bleomycin, consistent with previously reported replacement of tissue-resident AM with monocyte derived AMs in this model (37, 38), we sought an acute model to assess how loss of GATA2 affects tissue-resident AM responses. IL-33 binds to the ST2/IL-1 receptor accessory protein (IL-1RAP) heterodimer and has been linked to the pathogenesis of pulmonary fibrosis (39). Primarily expressed by type II alveolar epithelial cells and fibroblasts in the lung, IL-33 drives the expression of type 2 cytokines (e.g., IL-4, IL-5, and IL-13) by type 2 innate lymphoid cells, basophils, and mast cells, promoting a sustained inflammatory state with increased airway remodeling and obstruction (39–43). Intranasal IL-33 is sufficient to induce rapid and robust type 2 inflammation, characterized by eosinophilia and mucus hyperplasia (44).

**Figure 4:**
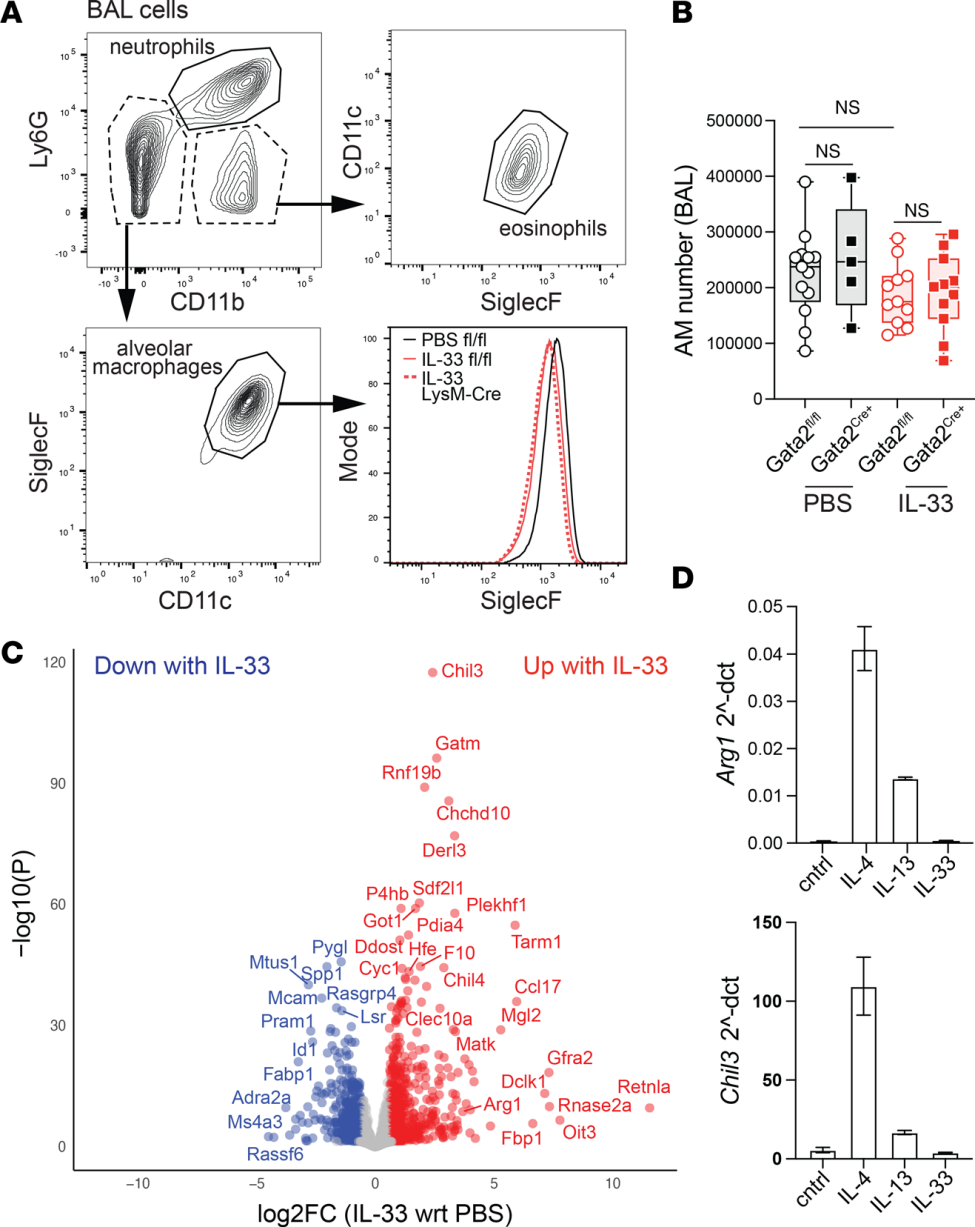
IL-33 indirectly induces a robust alveolar macrophage type 2 transcriptomic program. Mice were treated intranasally with 1 μg of IL-33 or PBS and euthanized 24 hours later for analysis. BAL was isolated from PBS- and IL-33–treated GATA2^fl/fl^ and GATA2^LysM-Cre^ mice; data points represent individual mice pooled from more than 3 independent experiments. (**A**) Representative BAL flow cytometry plots showing gating strategies for neutrophils, eosinophils, and AMs, and histogram showing AM SiglecF expression in representative PBS- or IL-33–treated mice. (**B**) SiglecF^+^CD11c^+^CD11b^–^ AMs were quantified from BAL; points represent individual mice pooled from 3 independent experiments; significance was determined using 1-way ANOVA with post hoc pairwise testing using Šídák’s multiple-comparison test. (**C**) Volcano plot of IL-33 differentially expressed genes, showing the –log_10_ of adjusted *P* value and log_2_ expression fold-change in IL-33–treated AMs relative to PBS treatment. (**D**) AMs were isolated by BAL and treated ex vivo with IL-33 (20 ng/mL), IL-13 (20 ng/mL), or IL-4 (10 ng/mL) for 24 hours. Quantification of mRNA transcripts for type 2 response genes *Arg1* and *Chil3* (*n* = 3; mean ± SEM) is representative of 2 independent experiments.

To assess acute, tissue-resident AM responses to profibrotic signals, we used a model of IL-33–induced type 2 inflammation. Mice were treated with IL-33 (1 μg) or PBS, and BAL cells were analyzed by flow cytometry. After 24 hours, AMs maintained high levels of SiglecF, and there was no significant change in AM numbers between PBS- and IL-33–treated mice (Figure 4, A and B). This suggests that, in contrast to bleomycin-induced fibrosis, the tissue-resident AM population remains intact 24 hours after IL-33 administration. CD11b^–^SiglecF^+^CD11c^+^ AMs were sorted from 8-week-old mice treated with 1 μg of IL-33 or PBS for RNA-seq analysis. Compared with control PBS-treated mice, intranasal IL-33 treatment resulted in 1,007 upregulated and 1,031 downregulated differentially expressed genes (DEGs) in AMs (Figure 4C), with upregulated genes including *Chil3*, *Arg1*, and *Retnla*, which are commonly associated with type 2 macrophage transcriptional programming (45). Kyoto Encyclopedia of Genes and Genomes (KEGG) pathway enrichment analysis of AM IL-33 DEGs highlighted TCA cycle, oxidative phosphorylation, and metabolic pathways as enriched in IL-33–induced inflammation (Supplemental Figure 3A).

Given that AMs do not highly express the IL-33 receptor ST2, we speculated that AM may not directly respond to IL-33, but rather to IL-33–induced type 2 cytokines. To explore this, we isolated AMs by BAL; treated them ex vivo with type 2 cytokines IL-4, IL-13, or IL-33; and then quantified *Arg1* and *Chil3* expression via quantitative PCR (qPCR). IL-4 and IL-13 stimulation resulted in a strong induction of *Arg1* and *Chil3* expression, whereas IL-33 stimulation did not elicit their expression (Figure 4D). Together, these data suggest that IL-33 rapidly induces type 2 inflammation in the lung, indirectly initiating a robust type 2 transcriptomic response in AMs. Our results are consistent with a recent report that IL-33 activates type 2 innate lymphoid cells to produce IL-13, which drives a type 2 inflammatory program in tissue-resident AMs (36).

### GATA2 restrains AM responses to type 2 inflammation.

**Figure 5:**
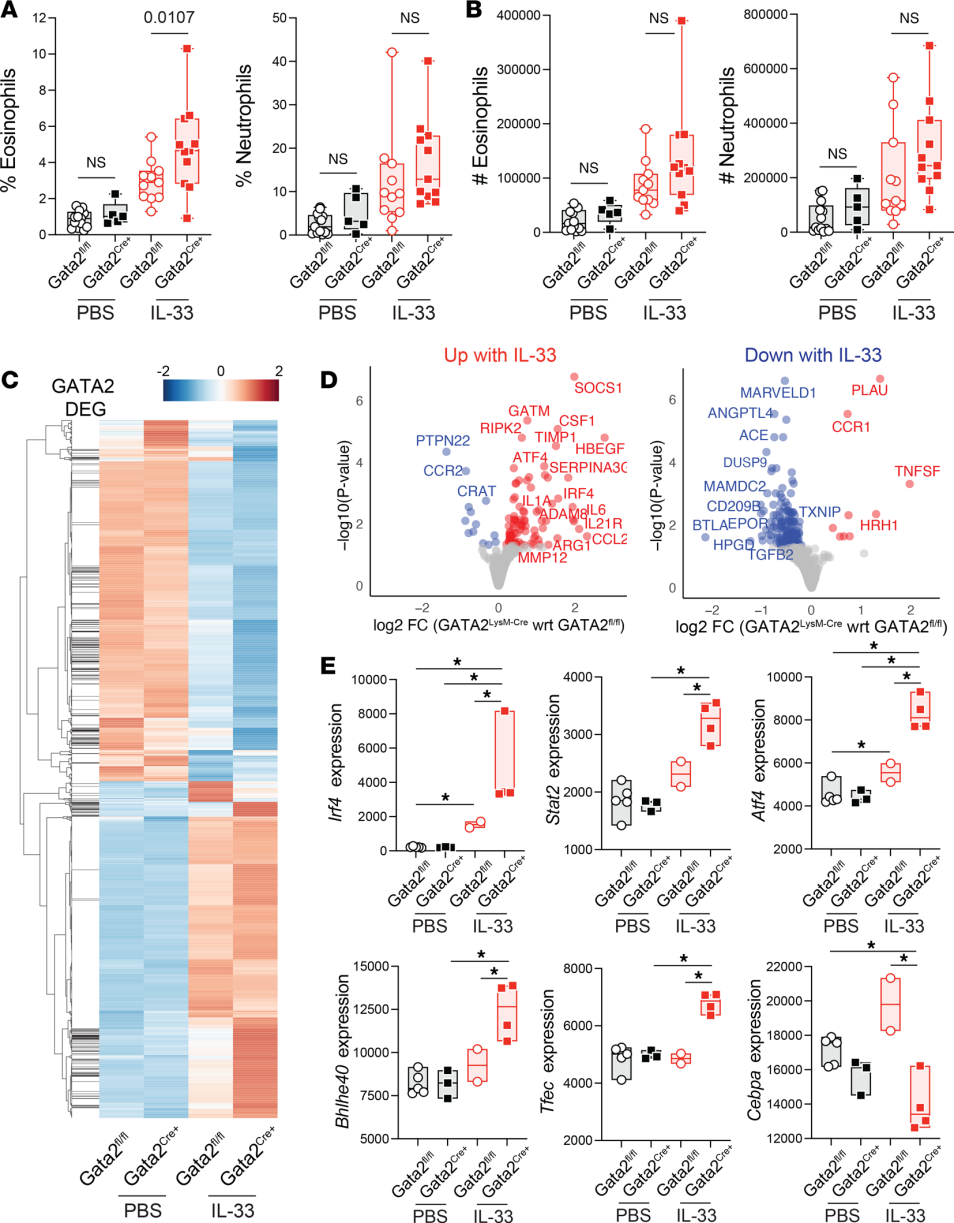
GATA2 restrains alveolar macrophage responses to type 2 inflammation. Mice were treated intranasally with 1 μg of IL-33 or PBS and euthanized 24 hours later for analysis. BAL was isolated from PBS- and IL-33–treated GATA2^fl/fl^ and GATA2^LysM-Cre^ mice, and the percentages (**A**) and numbers (**B**) of eosinophils and neutrophils were quantified by flow cytometry. Data points represent individual mice pooled from 3 independent experiments; significance was determined using 1-way ANOVA with post hoc pairwise testing using Šídák’s multiple-comparison test. (**C**–**E**) SiglecF^+^CD11c^+^CD11b^–^ AMs were isolated from BAL by FACS and analyzed by RNA-seq. (**C**) A heatmap showing *z*-scored expression of all IL-33 differentially expressed genes; genes differentially expressed with GATA2 deficiency are indicated in the column on the left. (**D**) Volcano plots of IL-33 upregulated genes (left) and downregulated genes (right), showing the –log_10_ of adjusted *P* value and log_2_ expression fold-change in GATA2-deficient AMs relative to GATA2^fl/fl^ AMs. (**E**) Normalized expression of differentially expressed transcription factor genes from RNA-seq analysis of BAL-isolated AMs across the indicated treatment groups; data points represent individual mice. Pairwise comparisons yielding statistically significant differential expression (adjusted *P* values < 0.05) are indicated with an asterisk.

To assess the effect of myeloid-specific GATA2 deficiency on type 2 inflammatory responses in the lung, we evaluated immune cell infiltration 24 hours after intranasal IL-33 administration. Flow cytometry analysis revealed a modest increase in the infiltration of eosinophils and neutrophils in the lungs of GATA2^LysM-Cre^ mice compared with GATA2^fl/fl^ controls, with the difference in eosinophil frequencies reaching statistical significance (Figure 5, A and B). To quantify the role of GATA2 in tissue-resident AMs during type 2 inflammation, we analyzed transcriptomic profiles of SiglecF^+^CD11c^+^CD11b^–^ AMs sorted from GATA2^fl/fl^ and GATA2^LysM-Cre^ mice 24 hours after intranasal IL-33 administration and identified 441 GATA2-dependent DEGs.

To elucidate the impact of GATA2 deficiency on AM expression of IL-33–regulated genes, we plotted the expression of the 2,038 IL-33–regulated AM genes across the 4 treatment groups (Figure 5C). We then directly compared AMs from IL-33–treated GATA2^LysM-Cre^ mice with those from IL-33–treated GATA2^fl/fl^ mice. This analysis revealed that GATA2 deficiency amplified inflammatory gene regulation, with IL-33–upregulated genes showing even higher expression and IL-33–downregulated genes showing even lower expression in GATA2-deficient AMs (Figure 5D). Specific examples include *Atf4*, *Mmp12*, *Timp1*, *Adam8*, *Il6*, and *Il1a*, which have been shown to promote lung fibrosis (46, 47); these genes were highly expressed in GATA2^fl/fl^ AMs treated after IL-33 administration, and these genes exhibited increased expression with GATA2 deficiency (Figure 5D). These data suggest that GATA2 deficiency results in enhanced AM transcriptomic responses to type 2 inflammation.

Analysis of differentially regulated TF genes (Figure 5E) showed that in response to IL-33–induced inflammation, GATA2-deficient AMs have increased expression of proinflammatory TF genes *Stat2*, *Irf4*, and *Atf4* compared with their WT littermates (Figure 5E). With GATA2 deficiency, we also noted differential expression of TFs and other genes that were not differentially expressed when comparing IL-33 with PBS in GATA2^fl/fl^ control mice (Figure 5E and Supplemental Figure 3B). Both *Bhlhe40* and *Tfec* have been previously associated with macrophage type 2 responses (48, 49). Notably, CEBPα is a known regulator of mitochondrial genes, its activity results in increased mitochondrial mass and respiration (24, 25), and its regulation in IL-33–induced inflammation is dependent on GATA2. This differential TF expression pattern is expected to drive enhanced proinflammatory functions in GATA2^LysM-Cre^ AMs and is consistent with the elevated type 2 inflammatory transcriptomic profile.

### GATA2 controls AM metabolic activity.

**Figure 6:**
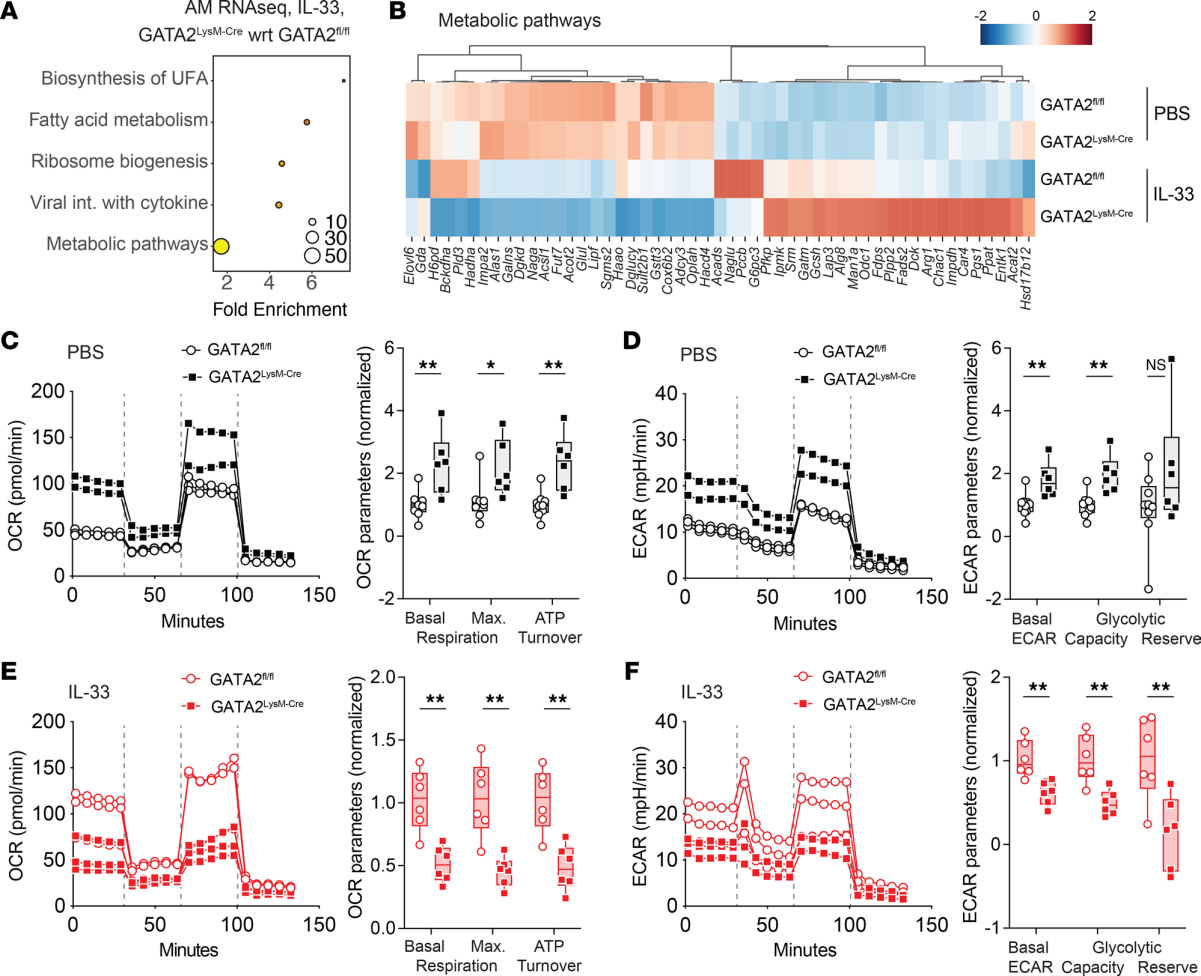
GATA2 controls alveolar macrophage metabolic activity. Mice were treated intranasally with 1 μg of IL-33 or PBS and euthanized 24 hours later for analysis. (**A** and **B**) SiglecF^+^CD11c^+^CD11b^–^ AMs were isolated from BAL by FACS and analyzed by RNA-seq. (**A**) KEGG pathway–focused gene set enrichment analysis using differentially expressed genes (DEGs) comparing GATA2^fl/fl^ and GATA2^LysM-Cre^ AMs from IL-33–treated mice. (**B**) Heatmap showing *z*-scored expression of GATA2 DEGs from the KEGG “Metabolic Pathways” gene set. (**C**–**F**) SiglecF^+^CD11c^+^CD11b^–^ AMs were isolated from BAL by FACS and plated for Seahorse extracellular flux analysis of AMs isolated from PBS-treated (black; **C** and **D**) or IL-33–treated (red; **E** and **F**) GATA2^fl/fl^ and GATA2^LysM-Cre^ mice. (**C**–**F**) OCR and ECAR kinetic traces show individual mice from 1 representative experiment where the vertical dashed lines represent sequential injections of oligomycin, FCCP, and rotenone/antimycin A, respectively. OCR and ECAR parameter plots show individual mice pooled from 3 independent experiments and normalized to the average of the GATA2^fl/fl^ group from each experiment. Significance was calculated using multiple unpaired 2-tailed *t* tests, with a 2-stage step-up (Benjamini, Krieger, and Yekutieli) and an FDR of 1%; ns = *P* > 0.05, *P < 0.05, **P < 0.01.

To elucidate GATA2-dependent mechanisms, we analyzed all GATA2 DEGs using unbiased KEGG pathway–focused gene set enrichment analysis and found that genes associated with metabolic pathways, fatty acid metabolism, and biosynthesis of unsaturated fatty acids were highly enriched (Figure 6A). Considering that AM lipid metabolism is central to their tissue-specific function of surfactant catabolism, we further explored the DEGs in the KEGG metabolic pathway gene set (Figure 6B). Although these genes were not differentially expressed at steady state, IL-33–induced metabolic reprogramming was more pronounced in GATA2-deficient AMs. We observed upregulation of genes associated with biosynthetic functions (*Impdh*, *Odc1*, and *Gatm*) (50–52) and stress adaption (*Chac1*, *Lap3*, and *GatmA*) (50, 53, 54), along with downregulation of genes required for mitochondrial function (*Cox6b2* and *Hadha*) (55, 56) and lipid processing (*Dgkd* and *Lipf*). These differential gene expression patterns suggest that GATA2^LysM-Cre^ AMs are more bioenergetically dysregulated in the context of type 2 inflammation (Figure 6B).

To directly address the impact of GATA2 deficiency on AM metabolic capacity, we performed Seahorse metabolic flux analysis on GATA2^fl/fl^ and GATA2^LysM-Cre^ AMs isolated under steady-state conditions. We found that GATA2-deficient AMs exhibited a statistically significant increase in basal respiration, maximal respiration, and ATP turnover compared with GATA2^fl/fl^ AMs (Figure 6C). We also measured extracellular acidification rates (ECARs), which revealed that GATA2-deficient AMs had increased basal ECAR and glycolytic capacity compared with GATA2^fl/fl^ AMs (Figure 6). Together, these data indicate that GATA2-deficient AMs are more metabolically active in the steady-state lung. We also performed this Seahorse assay after intranasal IL-33 treatment to GATA2^fl/fl^ and GATA2^LysM-Cre^ AMs at the time point corresponding to our RNA-seq analysis (24 hours) to assess how GATA2 affects metabolic flux of AMs in the context of type 2 inflammation. In contrast to our steady-state results, 24 hours after IL-33 treatment, mitochondrial respiration (oxygen consumption rate, OCR) and glycolytic capacity (ECAR) were decreased in GATA2^LysM-Cre^ AMs compared with GATA2^fl/fl^ AMs (Figure 6, E and F). Thus, our findings suggest that GATA2-deficient AMs exhibit mitochondrial dysfunction and implicate GATA2 in shaping the tissue-specific metabolic function of AMs.

We used the ChEA3 ChIP-Seq library to identify TFs whose targets are enriched in GATA2 DEGs (Supplemental Figure 3C). In addition to PPARγ and PU.1 (*Spi1*), several enriched TFs have been described to interact with GATA2 in various cellular contexts, including lineage commitment and cancer progression (Supplemental Figure 3C; darker bars). Focusing on top-ranked TFs by *P* value that are also highly expressed in AMs, we visualized these TFs and GATA2 alongside their target genes that were also metabolism-associated GATA2 DEGs (Supplemental Figure 3D). Notably, GATA2 appears closely linked to PU.1, consistent with known physical interactions between GATA2 and PU.1 and their context-specific cooperative and/or antagonistic interactions (27). We speculate that GATA2 interacts with TFs that are central to AM lipid metabolism and homeostatic functions, such PPARγ, the CEBP family, and PU.1, as has been demonstrated in other cell types (20, 21, 57, 58).

## Discussion

Regulatory circuits composed of cytokines and TFs dynamically shape macrophage function, allowing for functional plasticity that balances host defense and prevention of inflammation-associated tissue damage. Although AMs can mount inflammatory responses, the magnitude of these responses is dampened compared with monocyte-derived AMs responding to the same stimuli (4, 5), and the limited inflammatory function of AMs has been linked to their specialized metabolism (7, 8). Our results suggest that GATA2 contributes to the constrained inflammatory and metabolic plasticity of AMs, and that loss of its function leads to enhanced responses to type 2 inflammation.

Members of the GATA family (GATA1–GATA6) are known to antagonize or cooperate with other TFs to support cellular decisions, including directing cell differentiation, development, and function in diverse cell types such as T cells, mesodermal lineages, adipocytes, peritoneal macrophages, and blood cells (59). Notably, GATA3 and GATA6 play pivotal roles in regulating macrophage metabolism and inflammatory responses in a context-specific manner. GATA3 modulates the balance between inflammatory states in alternatively activated macrophages, and GATA6 maintains the identity and function of tissue-resident macrophages in the peritoneal cavity through regulation of genes controlling proliferation, survival, lipid metabolism, and responsiveness to stimuli (31, 60, 61).

Lung-specific expression of GATA2 in macrophages is consistent with the importance of GM-CSF in lung and AM homeostasis and with our observation that GM-CSF is sufficient to upregulate the expression of *Gata2* in BMDMs. GM-CSF regulation of GATA2 likely occurs via STAT5, given that STAT5 has been shown to directly bind the *Gata2* gene promoter and intronic region, along with the STAT5/GATA2 pathway being important for basophil and mast cell differentiation (16). Of note, GATA2 expression has also been described in macrophages isolated from atherosclerotic lesions, another context in which GM-CSF–activated STAT5 has been implicated in shaping macrophage functions (14, 29). GM-CSF–dependent regulation of GATA2 in AMs is particularly interesting considering known interactions between GATA2 and the GM-CSF–regulated TFs PU.1 and PPARγ in other cellular contexts. Given that these TFs are critical for the maintenance and homeostatic lipid-processing functions of AMs, we were surprised to find that myeloid-specific deletion of GATA2 did not reduce the number of AMs or alter their lipid content, uptake, or processing.

Known GATA2-interacting TFs shape homeostatic functions of AMs and also regulate macrophage inflammatory and metabolic reprogramming in response to stimuli. A ChIP-Seq library–based analysis identified TFs whose known targets were enriched in GATA2-dependent genes, with STAT5, PPARγ, PU.1 (*Spi1*), RUNX1, and MYC also being highly expressed in AMs. GATA2 has been reported to physically interact with PU.1, MYC, and RUNX1 (27, 62, 63), and we speculate that it shapes the activity of these TFs, serving to regulate metabolic and type 2 inflammatory functions in AMs. As such, with GATA2 deficiency, AMs show enhanced inflammatory responses and dysregulated metabolism, losing the hyporesponsiveness that is typically associated with appropriate AM behavior.

GATA2-deficient AMs exhibited increased metabolic potential at steady state, with both OCR and ECAR basal respiration rates being higher than those of GATA2^fl/fl^ AMs. In contrast, we found that GATA2 deficiency was associated with diminished ECAR and OCR rates in AMs exposed to type 2 inflammation for 24 hours. These results suggest a concurrent decrease in both glycolysis and oxidative phosphorylation, which suggests that loss of GATA2 compromises the ability of AMs to sustain coordinated glycolytic and oxidative metabolism in response to inflammatory stimulation. Coincident with decreased mitochondrial respiratory rates in the absence of GATA2, GATA2-deficient AMs also had decreased expression of mitochondrial protective genes such as *Ucp2* and *Epor*; oxidative stress regulators like *Txnip*; and antioxidant regulators *Glul*, *H6pd*, and *Dglucy*. The decreased expression of *Cepba*, a known transcriptional regulator of mitochondrial genes, may also be mechanistically linked to mitochondrial dysfunction in GATA2-deficient AMs. Considering these findings, we speculate that these metabolic signatures reflect a shift to an overactive but metabolically fragile macrophage state characterized by impaired mitochondrial support and maladaptive energy utilization during inflammatory challenge. Although we did not directly assess cellular exhaustion or mitochondrial damage at the functional level, the coordinated transcriptional and metabolic changes observed here are consistent with impaired metabolic adaptability under inflammatory stress. Thus, GATA2 has a role in regulating both the metabolic plasticity and inflammatory functions of AMs.

Metabolic dysregulation of AMs has been described in the context of chronic pulmonary diseases, including COPD, asthma, and pulmonary fibrosis (64, 65). Notably, diminished AM mitochondrial respiration and defective compensatory glycolysis reported in patients with COPD (66) are reminiscent of the mitochondrial dysfunction we observed in GATA2-deficient AMs during IL-33–induced inflammation. GATA2 deficiency was also associated with amplified AM type 2 inflammatory responses to acute stimulation, including increased expression of remodeling-associated genes (*Mmp12*, *Timp1*, *Hbefg*, and *Adam8*) (47, 67). Although our data do not support a role for myeloid-expressed GATA2 in driving bleomycin-induced pulmonary fibrosis, this mouse model is not well-suited to assess functions of GATA2 in tissue-resident AMs. In the bleomycin model, monocyte-derived AMs, rather than tissue-resident AMs, are the principal mediators of fibrosis pathology (5), and tissue-resident AM loss occurs within the first 3–7 days following bleomycin exposure (37, 38), preceding robust collagen accumulation, which emerges around day 14 and peaks between days 21 and 28 (68, 69). Interpretation is further complicated by LysM-Cre–mediated targeting of recruited myeloid populations, limiting conclusions regarding tissue-resident AM-intrinsic functions of GATA2 in this context.

In contrast to bleomycin-induced fibrosis, which resolves, asbestos-induced fibrosis persists for months, and genetic lineage–tracing approaches have been used to show that tissue-resident AMs are present within the fibrotic niche (70, 71). This model may therefore be suitable for interrogating the impact of the tissue-resident AM–intrinsic regulatory mechanisms described here. Considering recent evidence that tissue-resident AMs persist after induction of asthmatic inflammation with inhaled house dust mite (HDM) and exhibit similar transcriptomic responses in HDM- and IL-33–driven type 2 inflammation models (36), the HDM model provides an appropriate framework to examine how GATA2-dependent metabolic and transcriptional reprogramming of tissue-resident AMs shapes type 2 inflammatory responses to allergens.

Our findings reveal GATA2 as an important modulator of AM responsiveness that prevents aberrant transcriptional and metabolic reprogramming in response to type 2 stimuli. Given the critical role of known GATA2-interacting TFs in lung-specific AM functions, elucidating the interplay between GATA2 and these factors in AMs and the impact on target gene expression will enhance our understanding of the mechanisms controlling the metabolic and transcriptomic plasticity of AMs in response to changes in the microenvironment. These efforts will also provide mechanistic insight into GATA2 mutations and GATA2 downregulation associated with pulmonary diseases and disorders, and more generally, inform treatments for chronic pulmonary conditions worsened by AM pathological contributions.

## Methods

### Sex as a biological variable.

In vivo and ex vivo experiments were performed with age- and sex-matched male and female GATA2^fl/fl^ and GATA2^LysM-Cre^ mice. Reported phenotypes were observed in both male and female mice. Female mice were used for RNA-seq analyses of AM transcriptomic responses.

### Mice and in vivo treatments.

C57BL/6J (Jax stock 000664) mice were obtained from The Jackson Laboratory. GATA2^fl/fl^ (MMRRC stock 030290) mice were rederived from cryopreserved sperm and crossed to LyzM-Cre (Jax stock 004781) to generate myeloid-specific GATA2-KO (GATA2^LysM-Cre^) mice. Mice were bred to generate GATA2^LysM-Cre^ mice with a single copy of CRE and GATA2^fl/fl^ littermate controls. It has been previously demonstrated that the resulting KO allele has exon 5 deleted, which eliminates the DNA binding C-terminal zinc finger domain, resulting in a nonfunctional GATA2 protein (34). For lung infection, C57BL/6J mice were anesthetized with isoflurane and treated intranasally with 5 × 10^7^ *S*. *aureus* in 50 μL of PBS, then euthanized after 24 hours for BAL and cell processing for imaging. For in vivo IL-33 experiments, mice were anesthetized with isoflurane and administered 1 μg of IL-33 (BioLegend) in 50 μL of PBS or 50 μL of PBS intranasally and euthanized 24 hours later for subsequent processing and experimentation. To establish bleomycin-induced fibrosis, mice were treated intratracheally with bleomycin (Fresenius Kabi) at a dose of 3 U/kg and euthanized after 14 days for lung harvest and subsequent analysis.

### Isolation and stimulation of BMDMs.

Murine BM isolated from the femur and tibia was differentiated into BMDMs during a 6-day culture in complete DMEM with 10% FBS, 100 U/mL penicillin, 100 U/mL streptomycin, 2 mM L-glutamine, and 20 mM HEPES, supplemented with 60 ng/mL recombinant mouse M-CSF (R&D Systems). BMDMs were harvested on day 6 of the culture by scraping in cold PBS, replated in 48-well plates, and stimulated with 20 ng/mL of GM-CSF for 24 hours.

### Isolation, plating, and imaging of AMs.

Murine AMs were acquired by performing BAL using 1 mL of BAL buffer (PBS with 1% FBS and 0.5M EDTA), followed by 3–9 additional lavages. Cells were spun down at 400*g* for 7 minutes, red blood cells were lysed with ammonium-chloride-potassium (ACK) lysis buffer (Gibco), and remaining cells were resuspended in complete RPMI 1640 medium with 10% FBS, 2 mM L-glutamine, 100 U/mL penicillin, 100 U/mL streptomycin, and 100 mM sodium pyruvate. AMs were then plated at a density of 4,000–7,000 cells per well in 384-well black imaging plates (Griener Bio-One μClear) or approximately 40,000 cells per well in 96-well black imaging plates; rested for 30 minutes at 37°C, 5% CO_2_, and 95% relative humidity to facilitate adherence; and treated or stained as described. For infection experiments, after ACK lysis, CD11b^–^ cells were enriched using EasySep Mouse CD11b Positive Selection Kit II (STEMCELL Technologies), according to the manufacturer’s instructions. After plating or experimental treatment, cells were fixed with 4% paraformaldehyde (PFA) followed by permeabilization with 100% methanol, and then blocked with PBS containing 5% goat serum and 0.3% Triton X-100 for 1 hour at room temperature. Cells were stained overnight at 4°C with primary antibody diluted in PBS containing 1% BSA and 0.3% Triton X-100 using antibodies against CD11c (BioLegend, N418) or GATA2 (Invitrogen, PA1-100). Cells were then incubated with Cy3 goat anti-Armenian hamster IgG (Jackson ImmunoResearch, 127-165-099) and Alexa Fluor 488 goat anti-rabbit IgG (Invitrogen, A-11008) secondary antibodies. Cells were stained with Hoechst 3342 (Thermo Fisher Scientific), washed with PBS, and then imaged and analyzed with the CellInsight CX5 HCS platform and associated HCS Studio software (Thermo Fisher Scientific).

### BODIPY and DPPC uptake.

Isolated AMs were plated in starvation medium consisting of RPMI 1640 medium with 0.3% BSA. For BODIPY staining, 50 μL of a 2 μg/mL BODIPY (Thermo Fisher Scientific) solution prepared in prewarmed PBS was added to wells and incubated for 30 minutes. For DPPC uptake, after 30 minutes in starvation medium, AMs were treated with 20 ng/mL of NBD-DPPC (Avanti Research) diluted in pulmonary surfactant (CUROSURF porcine lung surfactant; 10% of the total volume) for 30 minutes. For both assays, AMs were washed with PBS, stained with Hoechst 3342 (Thermo Fisher Scientific), and imaged using the CellInsight CX5 HCS platform (Thermo Fisher Scientific). Spots were defined and quantified using HCS Studio software (Thermo Fisher Scientific).

### Lipid tracing with radiolabeled FFAs.

Sorted murine AMs were treated with radioactive FFAs (0.25 uCi ^3^H-oleic acid) in pulmonary surfactant (CUROSURF porcine lung surfactant; 10% of the total volume) diluted in RPMI 1640 medium with 0.3% BSA for 4 hours at 37°C with 5% CO_2_. At the end of incubation, cells were collected by centrifugation (400*g* for 10 minutes at 4°C), and lipids were Folch extracted (72). Cellular lipids were extracted overnight at 4°C in a 2:1 volume of chloroform/methanol. After centrifugation at 350*g* for 30 minutes at 4°C, the bottom clear layers were transferred to clean glass tubes and evaporated in a nitrogen evaporator (Organomation) in a waterbath set at 55°C. Dried lipids were resuspended in 200 μL to 1 mL of chloroform/methanol (2:1) to separate the lipid species by TLC. An aliquot was used for scintillation counting to determine total ^3^H-lipid levels, while another aliquot was loaded onto activated silica gel plates (Sigma-Aldrich). Lipids were fractionated using a solvent system (petroleum ether/diethyl ether/glacial acetic acid, 25:5:1 by volume). Lipid species and co-migrating lipid standards (Nu-Chek-Prep, Inc) were visualized with iodine vapor. Spots corresponding to standards for triglycerides, diacylglycerols, monoacylglycerols, FFAs, and phospholipids were scraped into scintillation vials and counted after adding scintillation fluid. The fraction of each lipid species was expressed as a percentage of total ^3^H-lipids loaded on activated silica gel plates.

### Phagocytosis.

Isolated murine AMs were plated in 384-well black imaging plates and rested for 30 minutes, at which time 200 μg/mL of pHrodo red *S*. *aureus* BioParticles (Invitrogen), Alexa Fluor 594 conjugate *S*. *aureus* BioParticles (Invitrogen) at 1:20 or 1 × 10^7^ polystyrene fluorescent yellow-green latex beads/mL (Sigma-Aldrich) were added to the appropriate wells and incubated for 90 minutes at 37°C. After incubation, media were discarded, and AMs were fixed with 4% PFA in PBS for 20 minutes at room temperature. For bead phagocytosis samples, AMs were blocked with antibody blocking buffer (5% goat serum with 0.3% Triton X-100 in PBS) for 1 hour and stained overnight with CD11c (BioLegend, clone N418), followed by Cy3 goat anti-Armenian hamster IgG secondary antibody (Jackson ImmunoResearch, 127-165-099). For pHrodo red and Alexa Fluor 594 *S*. *aureus* phagocytosis, cells were counterstained with nuclear stain Hoechst 3342 (Thermo Fisher Scientific). Cells were imaged using the CellInsight CX5 HCS platform and images were analyzed with HCS Studio software (Thermo Fisher Scientific). Latex beads, pHrodo red dye, and Alexa Fluor 594 *S*. *aureus* BioParticles were quantified using spot total intensities and spot total area to determine the degree of phagocytosis.

### Tissue preparation, flow cytometry, and cell sorting.

For AM sorting and quantification of cellular infiltrates, BAL-isolated cells were stained with antibodies recognizing CD11b-allophycocyanin-Cy7 (BioLegend, M1/70), CD11c-BV421 (BioLegend, N418), SiglecF-PE (BD Pharmingen, E50-2440), Ly6G-FITC (Invitrogen, RB6-8C5), and Ly6C-PerCP-cyanin (Cy) 5.5 (BD Pharmingen, AL-21). Ly6G-CD11b-CD11c^+^, SiglecF^+^ AMs were sorted using BD FACSAria II (BD Biosciences) and collected directly in TRIzol LS Reagent (Ambion). For additional flow cytometry analysis of BAL, data were collected with a BD Fortessa (BD Biosciences). For bleomycin experiments, right inferior and postcaval lobes of lungs were collected in digestion buffer (complete DMEM media supplemented with 10 μg/mL Liberase and 10 U/mL DNase I) and cut into 1 mm^3^ pieces followed by 30 minutes of incubation at 37°C. Samples were further dissociated using the program “m_lung_02_01” on the gentleMACS dissociator (Miltenyi Biotec) twice. The tissue homogenates were then filtered through 70 μm filter, and RBCs were lysed with ACK lysis buffer (Gibco). Single-cell suspensions were then stained with antibodies recognizing CD45-BUV395 (BD Horizon, 30-F11), SiglecF-AF647 (BD Pharmingen, E50-2440), CD11b-allophycocyanin-Cy7 (BioLegend, M1/70), CD86-BUV563 (BD OptiBuild, GL1), CD11c-BV421 (BioLegend, N418), Ly6C-BV605 (BioLegend, HK1.4), Ly6G-FITC (Invitrogen, RB6-8C5), CX3CR1-BV785 (BioLegend, SA011F11), CD64-PE (BioLegend, X54-5/7.1), and F4/80-AF700 (BioLegend, BM8) for tissue phenotyping. For bleomycin experiments, flow cytometry data were collected on an Aurora spectral cytometer (Cytek). Data were analyzed using FlowJo software.

### Quantification of collagen and trichrome stain.

After bleomycin administration, right superior lobes of the lung were harvested and snap-frozen in liquid N_2_. Subsequently, the tissue was weighed, washed with PBS to remove excess blood, and cut into small pieces, and then collagen was quantified using the Biocolor Sircol assay, according to the manufacturer’s instructions. Optical density was measured at 556 nm using a SpectraMax i3 (Molecular Devices), and values were derived based on the standard curve (4-parameter logistic fit). For trichrome staining, the left lobes of the lungs were inflated with 1 mL of PFA, and after 24 hours of incubation in PFA, tissues were transferred to ethanol and submitted to the Rangos Histology Core Facility for further tissue processing, staining, and imaging. Trichrome staining was performed using a Masson trichrome kit (Epredia). In brief, paraffin-embedded tissues were deparaffinized and rehydrated in deionized water followed by fixation in Bouin’s solution overnight. Sections were stained with Weigert’s hematoxylin for 10 minutes, stained with Biebrich Scarlet-Acid Fuchsin solution for 6 minutes, incubated in phosphotungstic-phosphomolybdic acid solution for 5 minutes, and counterstained with aniline blue stain solution for 6 minutes. Sections were incubated with 1% acetic acid solution for 1 minute and dehydrated with 100% ethanol, followed by xylene wash and mounting for imaging.

### RNA extraction and qPCR analysis.

Murine BMDMs and AMs cultured ex vivo were lysed using TRIzol (Ambion). For RNA-seq data, AMs were sorted directly into TRIzol LS (Ambion). RNA was isolated with the Direct-zol RNA MicroPrep kit (Zymo Research), following the manufacturer’s protocol. Reverse transcription of RNA to cDNA was performed using qScript cDNA SuperMix (Quantabio). TaqMan gene expression assays were used to perform qPCR on an CFX96 Real-Time system (BioRad). qPCR analysis was conducted using the ΔΔCt method, comparing target genes with the endogenous control gene, *Gapdh*, for normalization.

### RNA-seq analysis.

RNA concentrations were determined using a Qubit FLEX fluorometer, and libraries were generated with the Illumina Stranded mRNA Library Prep kit following the manufacturer’s instructions. Briefly, 25 ng of input RNA was used per sample. After adapter ligation, 15 cycles of indexing PCR were performed using IDT for Illumina RNA UD indexes. Libraries were normalized and pooled to 2 nM by calculating the concentration based on the fragment size (bp) and the concentration (ng/μL) of the libraries. Sequencing was performed on an Illumina NextSeq 2000 using a P3 200 flow cell. The pooled library was loaded at 750 pM, and sequencing was carried out with read lengths of 2 × 58 bp, targeting 25 million reads per sample. Sequencing data were demultiplexed by the on-board Illumina DRAGEN FASTQ Generation software (version 3.10.12). The *Mus musculus* reference genome (mm10) was downloaded from the UCSC genome browser, and STAR (73) was used to align and generate raw read counts. DESeq2 (74) was employed for read normalization and differential gene expression analysis. To remove genes with low expression, the 90th percentile of log_2_ size factor–normalized expression values was computed for each gene across all samples, and genes with a 90th percentile expression below an empirically defined threshold of 2.5 were excluded from further analysis. *P* values for differential expression were adjusted using the Benjamini-Hochberg method to correct for multiple hypothesis testing, and genes with adjusted *P* values below 0.05 were considered differentially expressed. RNA-seq heatmaps were generated with hierarchical clustering based on correlation distance and complete linkage. Data are available in NCBI’s Gene Expression Omnibus (GEO GSE298755). Gene set enrichment analysis was performed using ShinyGO 0.77 (75), leveraging the KEGG pathway database. “Alveolar Macrophage” and “Pulmonary Fibrosis” gene sets were taken from Harmonizome 3.0, from the “TISSUES Text-mining Tissue Protein Expression Evidence Scores” using normal human tissues and “CTD Gene-Disease Associations” datasets, respectively. All TF targets were identified using the ChEA TF target database, with the ChEA3 ChIP-Seq library being used to rank enriched TFs in Figure 6.

### Seahorse metabolic flux.

AMs were isolated from BAL and, in the case of IL-33–treated mice, Ly6G^–^CD11b^–^CD11c^+^SiglecF^+^ AMs were sorted as described above. Sorted and isolated AMs were analyzed for mitochondrial respiration using an XFe96 Extracellular Flux analyzer (Seahorse Bioscience). On the day of the experiment, all chemicals (e.g., oligomycin 2 μM, carbonyl cyanide-p-trifluoromethoxyphenylhydrazone [FCCP] 2 μM, and rotenone/antimycin A 0.5 μM) were prepared in assay medium, consisting of Seahorse XF base medium supplemented with 2 mM L-glutamine, 1 mM sodium pyruvate, and 10 mM glucose (without FBS or antibiotics), per the manufacturer’s recommendation. The machine was calibrated using calibrant buffer in the calibrant plate prior to the assay. AMs were seeded at a density of 80,000 cells per well in 100 μL of complete RPMI 1640 medium supplemented with 5% FBS, 1% L-glutamine, and 1% penicillin-streptomycin in poly-D-lysine–coated Seahorse XFe96 cell culture microplates and allowed to adhere for 1 hour at 37°C in a humidified incubator with 5% CO_2_. After adherence, cells were washed with assay medium, and 180 μL of fresh assay medium was added per well. Plates were incubated at 37°C in a non-CO_2_ incubator for 45 minutes prior to measurement. OCR and ECAR were measured at baseline and after sequential injections of oligomycin, FCCP, and rotenone/antimycin A. Each measurement cycle consisted of 3 minutes of mixing, 3 minutes of waiting, and 3 minutes of measuring. After each injection, 3 to 5 measurement cycles were performed. All experimental groups were run on the same plate on the same day. ATP turnover was calculated as the difference between the last rate measurement before the oligomycin injection and the minimum measurement after oligomycin injection. Maximal respiration was defined as the highest OCR measured following FCCP injection, minus non-mitochondrial respiration. Spare respiratory capacity was calculated by subtracting basal respiration from maximal respiration. ECAR values were measured in the same well, with an optimal glucose level of 10 mM. The basal ECAR value was defined as the measurement obtained immediately before oligomycin injection. In the classical glycolytic assay procedure (glucose-free media), the final concentration of glucose added to the port was 10 mM while measuring flux. The basal ECAR value in this classical method was calculated by subtracting the last rate measurement before the glucose injection from the maximum rate measurement before the oligomycin injection, which essentially provided the same value as calculated by the method described here. Glycolytic capacity was defined as the rate measured after the oligomycin injection. Glycolytic reserve was calculated as the difference between glycolytic capacity and the basal ECAR value.

### Statistics.

Statistical analysis was performed using GraphPad Prism software. For most experiments, an unpaired 2-tailed *t* test or 1-way ANOVA with post hoc pairwise testing using Šídák’s multiple-comparison test was used, and a *P* value of 0.05 was considered statistically significant. For analysis of Seahorse OCR and ECAR parameters, significance was calculated using multiple unpaired 2-tailed *t* tests, with a 2-stage step-up (Benjamini, Krieger, and Yekutieli) and an FDR of 1%. Statistical analysis of RNA-seq data is described above. Error bars show range, minimum to maximum, unless otherwise noted in the figure legend.

### Study approval.

Animal studies were reviewed and approved by the IACUC of the University of Pittsburgh and were conducted in accordance with institutional guidelines and NIH policies for the care and use of laboratory animals.

### Data availability.

The RNA-seq data reported in this paper have been deposited in GEO (GSE298755). All other data needed to evaluate the conclusions in the paper are present in the paper or in the supplemental materials. The code used in this study is available at https://github.com/gottschalklab/GATA2 (commit ID 8716443). Individual data values shown in graphs are available in the Supporting Data Values file.

## Author contributions

MJS, SG, AF, JFA, ABK, and RAG were responsible for the experimental and analytical strategy. MJS, SG, FR, JK, BAM, LT, OSB, KES, AK, KB, A Suleyman, A Sekyere, MH, VS, and AK conducted experiments. MJS, SG, AF, JK, SK, and RAG performed analysis and visualization. MJS, SG, AF, NN, JFA, ABK, and RAG interpreted the results. MJS, SG, AF, and RAG wrote the original draft. GMD, NN, JFA, ABK, and RAG reviewed and edited the manuscript. Co–first authors are listed in reverse alphabetical order.

## Funding support

This work is the result of NIH funding and is subject to the NIH Public Access Policy. Through acceptance of this federal funding, the NIH has been given a right to make the work publicly available in PubMed Central.

- NIH/National Heart, Lung, and Blood Institute (NHLBI) grant R01HL162658 (to RAG).
- NIH/NHLBI supplement R01HL162658-02S1 (to MJS).
- NIH/National Institute of Allergy and Infectious Diseases training grant 5T32-AI1089443 (to SK).

## Supplementary Material

Supplemental data

Supporting data values

## Version 1 02/19/2026

In-Press Preview

## Version 2 04/08/2026

Electronic publication
