# Supplementary material for: GATA2 controls alveolar macrophage inflammatory gene expression and metabolic function
Source: JCI Insight. 2026 Feb 19;11(7):e196246. doi: 10.1172/jci.insight.196246 (PMC13134736; doi:10.1172/jci.insight.196246)
Supplement: Supplemental data [file jciinsight-11-196246-s096.pdf]

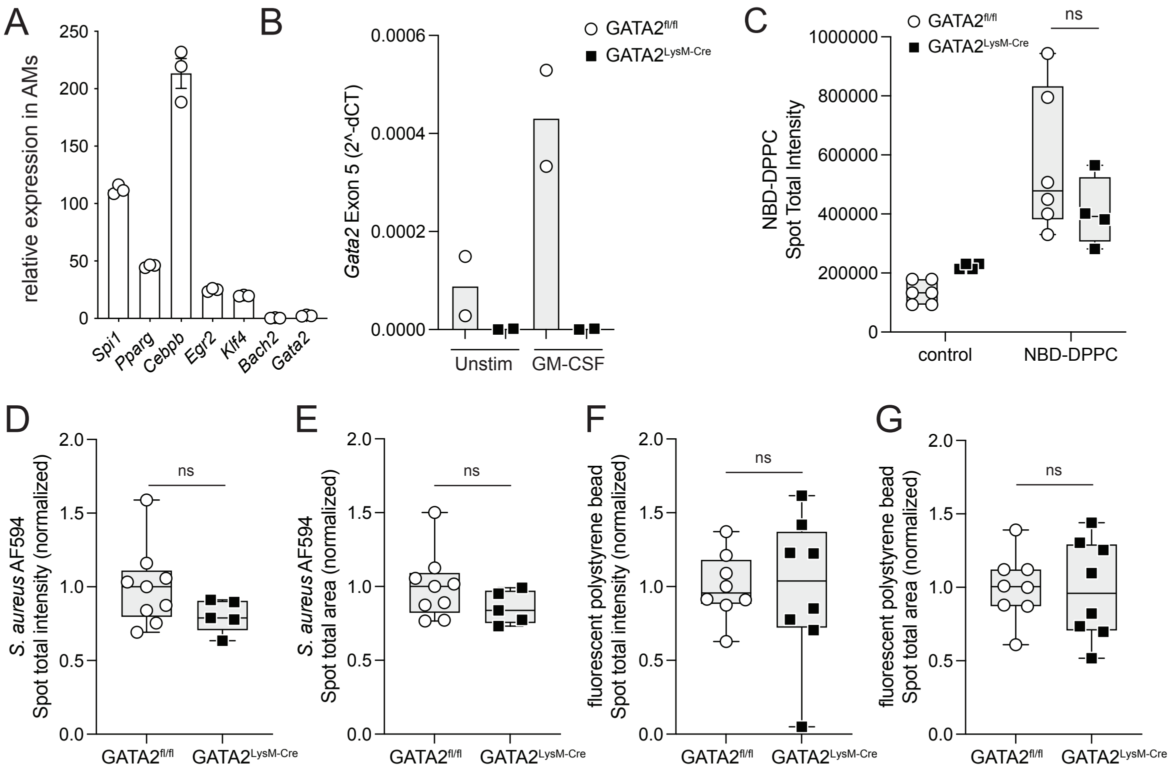

Supplemental Figure 1: Normal homeostatic functions in GATA2-deficient alveolar macrophages
(A) Re-analysis of RNA-seq data from Qie et al (PMID: 36450731), quantifying relative expression of transcription factor genes in tissue resident alvolear macrophages. (B) Bone marrow derived macrophages were differentiated from GATA2$^{fl/fl}$ and GATA2$^{LysM\text{-}Cre}$ mice and stimulated with GM-CSF for 24 hours and *Gata2* exon 5 was quantified by qPCR. (C-D) GATA2$^{fl/fl}$ and GATA2$^{LysM\text{-}Cre}$ AMs were isolated using bronchoalveolar lavage (BAL). (C) AMs were incubated with NBD-DPPC for 30 minutes and the spot total intensity of accumulated NBD-DPPC was quantified by immunofluorescence imaging. (D, E) AMs were incubated with Alexa Fluor 594 labeled *S. aureus* bioparticles for 90 minutes and the spot total intensity or area of internalized bioparticles was quantified via immunofluorescence imaging, as indicated. (F,G) AMs were incubated with fluorescent yellow-green polystyrene beads for 90 minutes and spot total intensity and spot total area of internalized beads were quantified via immunofluorescence imaging. For all plots, data points represent individual mice. Data in B are representative of two independent experiments, data in C-D were pooled from three independent experiments, data in E-F were pooled from four independent experiments. Significance was determined using unpaired t-test

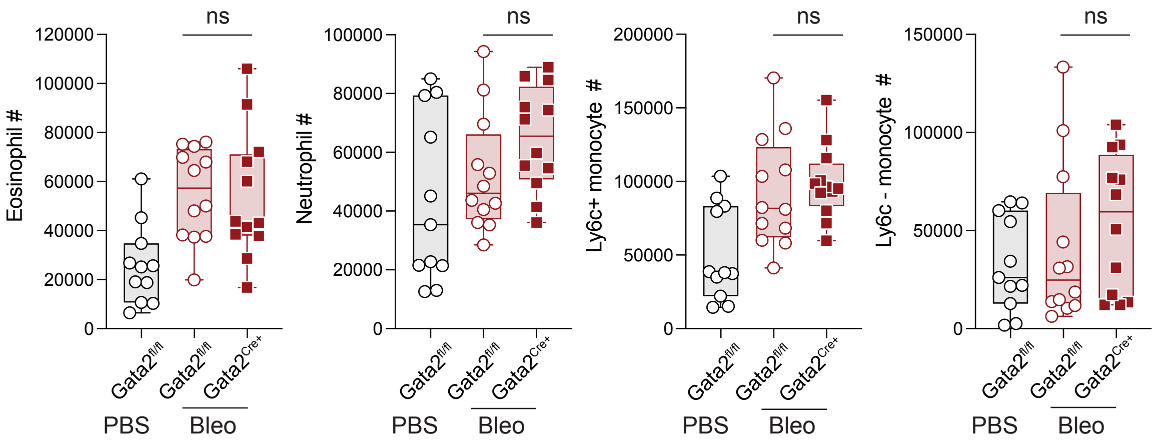

Supplemental Figure 2: Effects of myeloid-specific GATA2 deficiency on bleomycin-induced cellular infiltration.
Mice were treated intratracheally with bleomycin (3units/kg) and sacrificed 14 days later for lung tissue harvest and analysis. Quantification of cell populations by flow cytometry; gating strategy shown in Figure 3C. Points represent individual mice pooled from three independent experiments. Significance was determined using one-way ANOVA with post-hoc pairwise testing using Sidak's multiple comparison test.

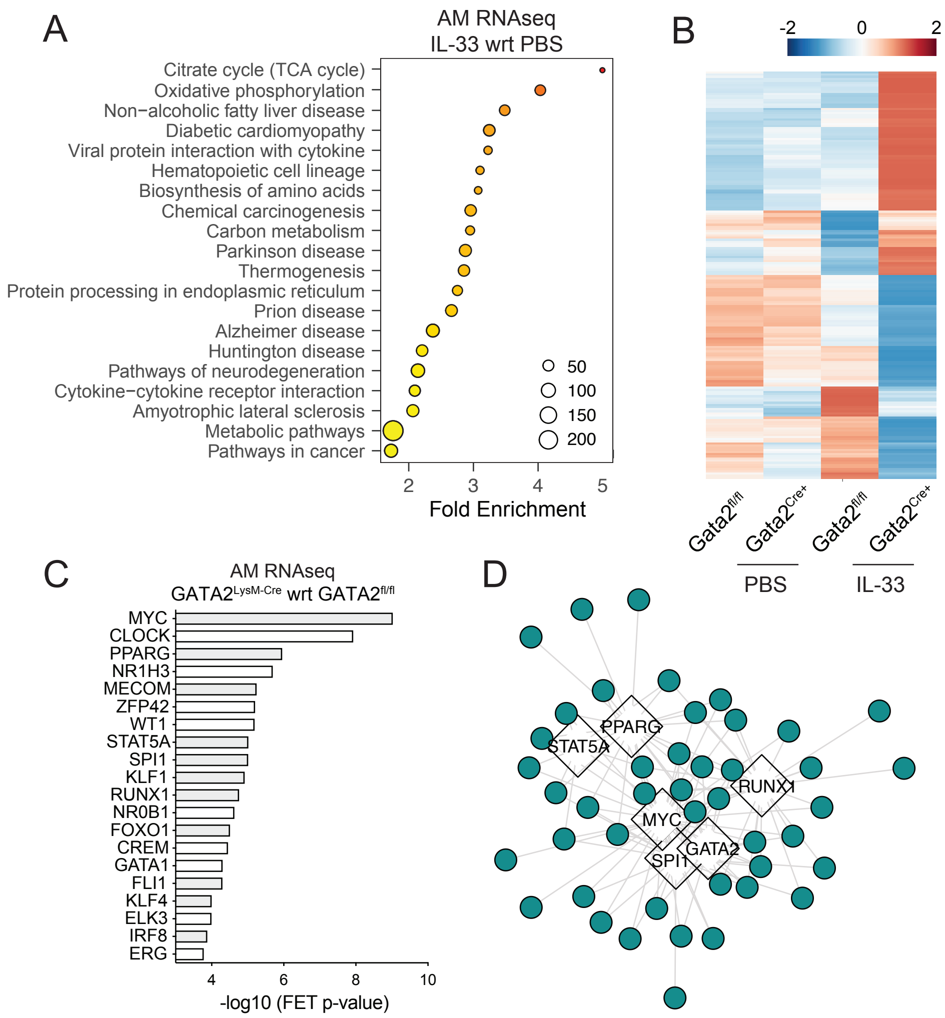

Supplemental Figure 3: GATA2-dependent regulation of alveolar macrophage gene expression. Mice were treated intranasally with 1ug of IL-33 or PBS and sacrificed 24 hours later for analysis. SiglecF$^+$CD11c$^+$CD11b$^-$ AMs were isolated from BAL by flow sorting and analyzed by RNA-seq. (A) KEGG pathway enrichment analysis of differentially expressed genes (DEGs) comparing AMs from IL-33-treated mice with AMs from PBS-treated mice. (B) Heatmap showing z score expression of GATA2 differentially expressed genes that were not differentially expressed between IL-33-treated and PBS-treated GATA2$^{fl/fl}$ mice. (C) ChIP-X Enrichment Analysis 3 (ChEA3; ChIP-seq library) analysis of transcription factor target enrichment within AM GATA2 DEGs; fishers exact test (FET). (D) Network visualization of transcription factors (diamond nodes) with high target enrichment from ChEA3 analysis and high expression in AMs, with known target genes that are also GATA2 DEGs from the KEGG “Metabolic Pathways” gene set (circle nodes); edges represent transcription factor-target interaction.
